# MRI-based classification of lateral hinge fractures in medial opening wedge high tibial osteotomy

**DOI:** 10.1186/s43019-026-00312-w

**Published:** 2026-03-03

**Authors:** Woon-Hwa Jung, Minish Katkar, Min-Seok Seo, Dong-Hyun Kim, Ryohei Takeuchi

**Affiliations:** 1 Department of Orthopaedic Surgery, Murup Hospital, 243, 3·15-daero, Masan, Changwon-si, Gyeongsangnam-do 51264 Republic of Korea; 2 Department of Orthopaedic Surgery, Saiwai Tsurumi Hospital, Tsurumi-ku, Yokohama, Japan

**Keywords:** Medial opening wedge, High tibial osteotomy (HTO), Lateral hinge fracture (LHF), Classification, Proximal tibiofibular joint, Delayed, Union time, Magnetic resonance imaging (MRI)

## Abstract

**Aim:**

To investigate the variants of lateral hinge fracture and its outcome, as well as to develop a MRI based classification on the fracture line pattern.

**Methods:**

This retrospective study analyzed 250 knees from 227 patients (169 females, 58 males) who underwent medial opening wedge high tibial osteotomy. Lateral hinge fractures were detected using MRI and classified into four types on the basis of the fracture line pattern: type A (proximal to the tibiofibular joint), type B (into the proximal tibiofibular joint), type C (distal to the tibiofibular joint), and type D (proximal into the joint). Patients were followed up with radiographs and computed tomography (CT) scans to monitor outcome.

**Results:**

Type A fractures had a shorter union time (3.66 months) than type B (5.17 months), type C (6.24 months), and type D (5.75 months). Type B had a delayed union rate of 20%, higher than that of type A (2.46%). Statistical analysis confirmed that type A fractures had significantly better outcomes than types B, C, and D. Type B fractures are by definition Takeuchi type I fractures but exhibit clinical characteristics similar to type II fractures, including longer union times and a higher risk of delayed union.

**Conclusions:**

Type A fracture has union rates similar to those in non-fracture groups, whereas type B fracture has clinical similarities to Takeuchi type II fractures and therefore should be considered and managed as a subtype of Takeuchi type II fractures.

## Introduction

Medial opening wedge high tibial osteotomy (MOWHTO) is a widely performed procedure for correcting varus malalignment in the knee. However, it is associated with a significant complication rate, with nearly a quarter of cases requiring additional intervention. One of the most common and concerning complications is lateral hinge fracture (LHF), which occurs when the lateral cortical hinge essential for maintaining stability during the osteotomy suffers an unintended break [1–3]. Studies have reported LHF incidence ranging from 3% to 34%, influenced by factors such as osteotomy location, hinge position, and gap width [4–11]. This complication is particularly problematic as it can lead to delayed union, nonunion, or implant failure due to reduced stability and increased micromotion at the osteotomy site.

Radiographic analysis by Takeuchi et al. revealed that lateral cortical hinge fractures in medial opening wedge high tibial osteotomy (MOWHTO) exhibit distinct patterns, each influencing the healing process differently and outcome of the surgery. On the basis of radiographs, fractures were categorized into three types: type I, where the fracture extends along the osteotomy line and remains proximal to or within the tibiofibular joint; type II, where the fracture extends to the distal portion of the proximal tibiofibular joint; and type III, involving a lateral tibial plateau fracture [1]. However, during a study on LHF detection using MRI, Jung et al. [12] discovered that certain Takeuchi type I fractures on radiographs exhibited a clinical prognosis similar to that of Takeuchi type II fractures. The study aims to investigate this variant of Takeuchi type I fracture and its outcome, as well as to develop an MRI classification based on the LHF line pattern. The study hypothesizes that there exists a variant/subtype of Takeuchi type I fracture that has an outcome similar to that of a Takeuchi type II fracture.

## Materials and methods

This retrospective, nonrandomized study reviewed 250 knees with varus deformities from 227 patients (169 females [74.6%], 58 males [25.4%]) treated with MOWHTO at our hospital. All surgeries, performed by a single surgeon, occurred between January 2014 and June 2017 or between January 2023 and January 2024. The affected side was nearly evenly distributed, with 131 cases (52%) on the right and 119 (48%) on the left. The average patient age was 60.4 ± 13.2 years (range 34–83 years). Patients underwent clinical and radiographic evaluations at 4 weeks, at 6 weeks, every month thereafter until 1 year, and annually thereafter.

### Inclusion criteria

Active patients with symptomatic medial osteoarthritis or articular cartilage lesions of the knee joint, varus limb malalignment, and an intact lateral joint compartment or cartilage lesions with an International Cartilage Repair Society (ICRS) grade of less than I.

### Exclusion criteria

Active knee infection, severe patellofemoral osteoarthritis, ≥ 10° anatomic varus alignment, flexion contracture over 15˚, and varus/valgus instability greater than 10˚ on a stress view with a Telos device (Telos, Marburg, Germany).

The study was approved by our institutional review boards, and informed consent was obtained. Medial opening-wedge HTOs were performed using the TomoFix™ plate (Synthes, Oberdorf, Switzerland).

### Surgical procedures

The Mikulicz mechanical axis was used to evaluate lower limb alignment and determine the required correction. HTO was performed to align the mechanical axis at 62% from the medial eminence, as described by Fujisawa et al. [13]. Arthroscopic examination was performed in all cases prior to MOW-HTO to assess the status of cartilage and the meniscus. Biplanar MOW-HTO, as described by A.E. Staubli, was carried out, maintaining the medial opening gap using a distractor [7]. The gap was filled with β-TCP (chronOS^®^ wedge, semicircular; Synthes, Solothurn, Switzerland) and autologous distal femur bone graft. The osteotomy was stabilized with a TomoFix™ plate. Weight-bearing axis correction was confirmed with a metallic ruler and intraoperative c-arm imaging. The bone graft-harvesting technique, a modified version of the Moyad et al. method [12], used Osteochondral Autograft Transfer System (OATS) donor cutting tubes to extract bone from the medial femoral condyle near the adductor tubercle through a 15-mm incision. This minimally invasive technique resulted in only a minimal increase in operative time.

### Postoperative rehabilitation

Early mobilization without weight-bearing with active straight leg raising, passive range of motion exercises, continuous passive motion, and muscle strengthening starting 1 day after surgery. Range-of-motion exercises continued until a maximum flexion of 130° or more was achieved within 3 weeks. Patients were non-weight-bearing for 4 weeks, after which partial weight-bearing with a walker was initiated. Full weight-bearing with a cane began once callus formation reached zone 2, as defined by Jung et al. [17], depending on the patient’s tolerance.

### Radiologic evaluation

A 1.5-T multiplanar MRI was performed on postoperative day 2, and radiographs and CT scans were evaluated during follow-up visits. We assessed MRI in the sagittal and coronal views of the lateral zone of the tibia. The lateral zone is a bony bridge located laterally to hinge line (an anteroposterior line tangent to the medial edge of the fibular head) on the axial plane of the CT scan [14]. A fracture was defined on MRI if at least one cut section of the lateral zone showed a linear low-signal intensity (SI) surrounded by an intermediate-SI area on T1WI and the linear low SI surrounded by high SI on T2WI, extending from the endpoint of the osteotomy to the lateral cortex. Fractures were diagnosed on CT scans by hypodense fracture lines in the cortex. The anteroposterior tibial slope view minimized parallax at the osteotomy site, ensuring full visibility of the osteotomy gap [15]. Loss of correction was measured by the hip–knee–ankle (HKA) angle on standing full-length radiographs at day 4 post-surgery and during the 4-week follow-up. Bone union was considered radiologically complete when bridging callus reached zone 3, with at least 50% of the osteotomy gap bridged with callus [16].

**Fig. 1:**
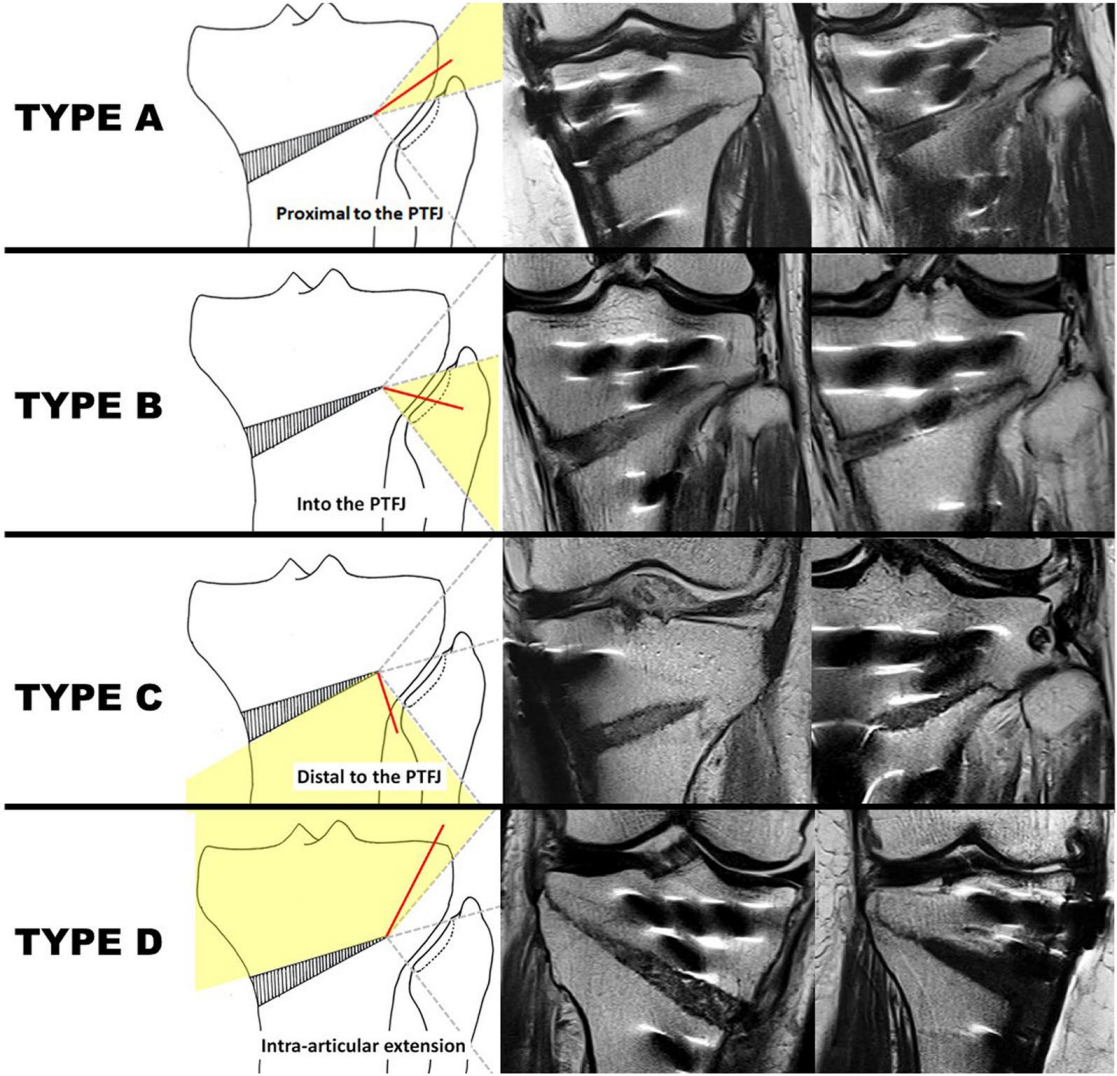
Examples of MRI-based classification (coronal cut sections). The red lines indicate the fracture line. In type A, the fracture occurs where the osteotomy line extends proximally to the tibiofibular joint without involving the joint itself. Type B refers to a fracture line that extends into the proximal tibiofibular joint. In type C, the fracture extends distal to the proximal tibiofibular joint. Finally, type D is characterized by a fracture that extends proximally into the joint

We have observed four distinct types of fractures around the lateral cortical hinge, classified on the basis of the fracture line pattern. The classification is as follows (Fig. 1):

- Type A: a fracture in which the osteotomy line extends proximally to the tibiofibular joint without involving the joint
- Type B: a fracture in which the osteotomy line extends into the proximal tibiofibular joint
- Type C: a fracture in which the osteotomy line extends distal to the proximal tibiofibular joint
- Type D: a fracture in which the osteotomy line extends proximally into the joint

Two orthopedic surgeons, who were not involved in the initial operation, assessed the fractures. They evaluated the interobserver reliability using Cohen’s kappa coefficient, which measured 0.82, 0.86, and 0.91 for type A, B, and C fractures, respectively. For type D fractures, both reviewers reached the same conclusion.

Data were analyzed using standard descriptive statistics (mean ± standard deviation, frequency, and percentage). Group comparisons of continuous variables—including age, body mass index (BMI), bone mineral density (BMD), osteotomy opening distance, and union time—were performed using one-way analysis of variance (ANOVA), followed by Tukey’s honestly significant difference (HSD) post hoc test for pairwise comparisons when significant differences were detected. Comparisons of categorical variables—including fracture type distribution, gender distribution, radiographic visibility of fractures, complication rates, and delayed union rates—were assessed using the chi-squared test. Union time differences among fracture types and the non-fracture group were evaluated using ANOVA, and delayed union proportions were compared using chi-squared analysis. A *p*-value < 0.05 was considered statistically significant for all tests.

## Results

This study includes a total of 250 knees among 227 patients, of whom 169 (74.6%) were female and 58 (25.4%) were male. The affected side was nearly evenly distributed, with 131 cases (52%) on the right side and 119 cases (48%) on the left side. The average patient age was 60.4 ± 13.2 years (range 34–83 years). The mean BMI was 24.46 ± 5.63 kg/m^2^ (range 20.4–35.7 kg/m^2^). Bone mineral density (BMD) values averaged −1.45 ± 1.73 (range −4.0 to 2.9). The average opening distance was 7.93 ± 2.98 mm (range 4.0–20.0 mm).

A total of 250 knees were analyzed: 149 exhibited fractures visible on MRI, while 101 showed no fractures. On the basis of the MRI fracture pattern, 81 were classified as type A, 41 as type B, 23 as type C, and 4 as type D. Out of the 149 fractures detected on MRI, only 93 were visible on follow-up radiographs and CT scans. These included 31 out of 81 (38.27%) type A fractures, 37 out of 41 (90.24%) type B fractures, 22 out of 23 (95.65%) type C fractures, and 3 out of 4 (75%) type D fractures.

### Union time

Type A had the shortest average union time of 3.66 months, followed by type B at 5.17 months, while type C had the longest average union time of 6.24 months. The nonfracture group demonstrated union time of 2.58 months (Table 1). The difference between type A and type B was found to be statistically significant (*p* = 0.0425). However, there was no significant difference between types B, C, and D, suggesting that type A had a significantly different union time compared with the other types. There was no statistical difference in union time between nonfracture group and type A fracture. It is also interesting to note that type B had a longer union time than type A, even though both are Takeuchi type I fractures by convention. Additionally, it is notable that type B had a union time similar to that of type C and type D.

**Table 1:** Types of lateral hinge fractures on MRI taken after MOWHTO and outcomes

Type	Average union time (months)	Delayed union cases	Delayed union percentage	Average opening distance (mm)	Loss of correction	Bone mineral density
A	3.66	2/81	2.46%	9.25	−0.65	−0.39
B	5.17	8/41	19.51%	8.60	−0.85	−0.63
C	6.24	5/23	21.73%	7.28	0.37	−0.53
D	5.75	1/4	25%	12.75	0.73	−0.45

### Delayed union

Type A had the lowest delayed union rate, with only 2.46% of cases experiencing delayed healing. Another observation is that the outcome of type B is poorer than type A in terms of union delay, with around 20% of type B cases showing significantly higher delayed union.

### Opening distance

Type A has a significantly lower opening distance than type B and C, with a difference of −1.64 (*p* = 0.033). Type D, on the other hand, has a significantly higher opening distance than both type A (difference of 3.83, *p* = 0.018) and type B (difference of 4.24, *p* = 0.009). The difference between type A and type D is particularly pronounced, with a highly significant difference of 5.47 (*p* = 0.0006), showing that type D has a significantly higher opening distance than type A. However, there was no significant difference between type B and type C (*p* = 0.247).

There was no statistically significant difference in BMD among the fracture types. Chi-squared test showed no statistically significant difference in gender distribution across the types. The chi-squared test revealed no significant differences in infection or complication rates among the types (Table 2).

**Table 2:** Statistical analysis of fracture types in the study

	*F*-statistic	*p*-Value	Conclusion	Tukey’s HSD test
Union time	3.68	0.014	Statistically significant difference	A versus B: significant; *Q* = 3.97 (*p* = 0.0323) A versus C: significant; *Q* = 6.43 (*p* = 0.00007) A versus D: significant; *Q* = 3.90 (*p* = 0.0335)
BMD	0.35	0.786	Not statistically significant	
Loss of correction	0.85	0.471	Not statistically significant	
Opening distance	6.14	0.0005	Statistically significant difference	Significant differences found between: Type A versus type C (*p* = 0.033) Type A versus type D (*p* = 0.018) Type B versus type D (*p* = 0.009) Type C versus type D (*p* = 0.0006) No significant differences found between: Type B versus type C (*p* = 0.247)

## Discussion

This is a novel study that classifies lateral hinge fractures in MOWHTO on the basis of MRI. In addition to having a superior detection rate for acute hinge fractures, the fracture pattern becomes evident on MRI much sooner than on radiographs. It was observed that type A and B had fracture line patterns similar to Takeuchi type I, while type C and D resembled the previously described Takeuchi type II and III, respectively. Nakamura et al. [18] reported that type I fractures had better outcomes than type II and III. The union time of type A fractures was found to be similar to the nonfracture group. It was also observed that, while only 38.27% of type A fractures detected on MRI became visible only on follow-up radiographs/CT scans, 90.24% of type B fractures detected on MRI became visible only on follow-up imaging. This was also found to be similar to 95.65% in type C fractures.

A difference in the incidence of delayed union was observed between type A and type B fractures, with type B fractures having approximately a 20% risk of delayed union, compared with just 2.46% in type A fractures. The similarities in the percentage of fractures becoming evident on follow-up radiology, union time, and delayed union highlight a resemblance between type B and type C fractures. This suggests that type B is a variant/subtype of Takeuchi type I, appearing radiographically similar to Takeuchi type I but clinically resembling Takeuchi type II fractures. This study, therefore, supports the need for a subclassification of type I fractures, leading to the development of distinct rehabilitation protocols. We recommend managing this variant/subtype (type B) with postoperative protocols similar to those for Takeuchi type II fractures to optimize surgical outcomes in MOWHTO.

**Fig. 2:**
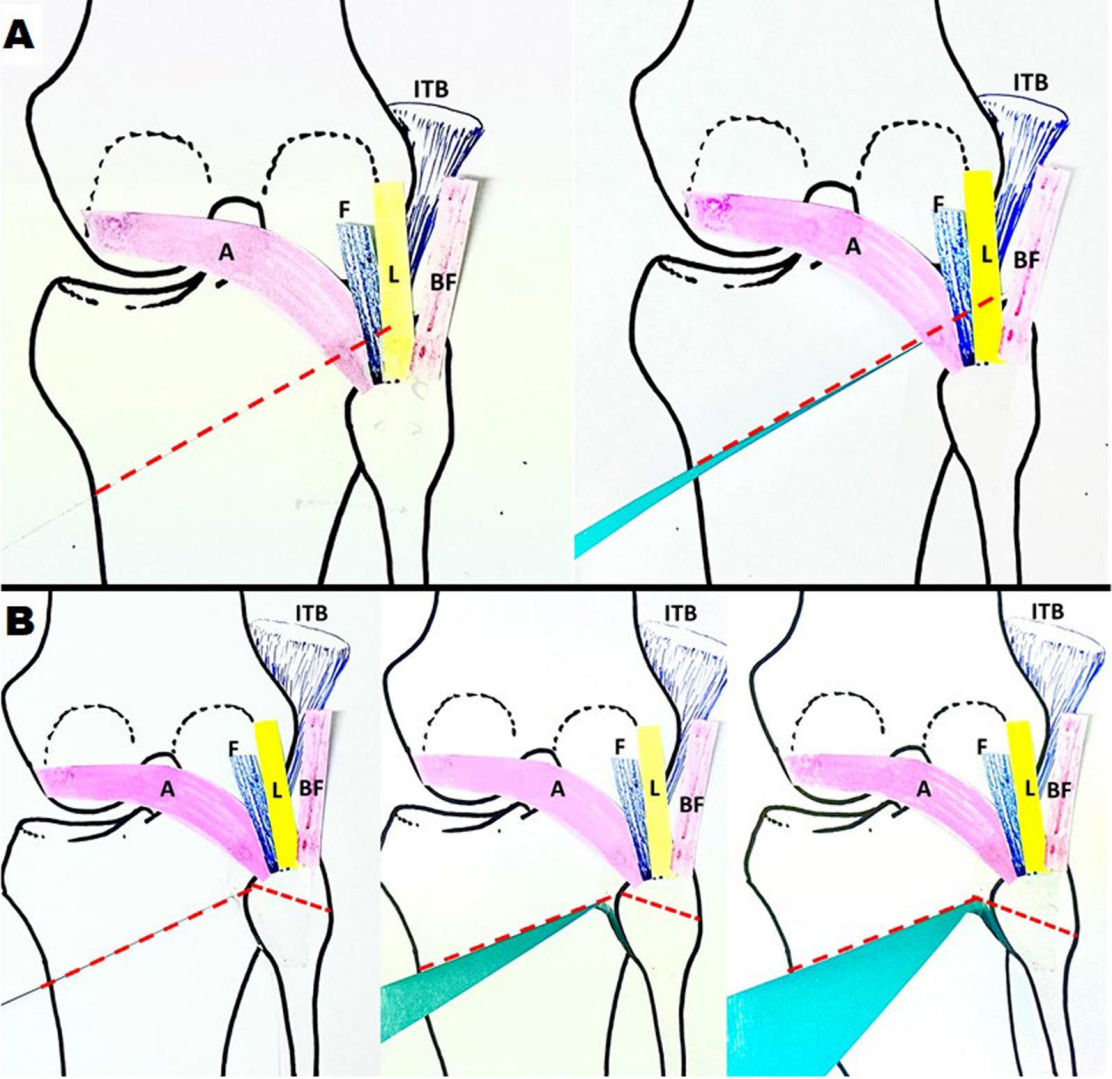
Stability of different fracture types in high tibial osteotomy. This figure illustrates the stability of different fracture types based on their anatomical location relative to supportive structures. **A** Type A fractures: the fracture line extends within the anterolateral and posterolateral structures, which provide additional support and stabilize the fracture. **B** Type B and C fractures: the fracture line is located distal to these supportive structures, resulting in instability due to the lack of reinforcement from key anatomical structures. ITB, iliotibial band; BF, biceps femoris, L, lateral collateral ligament, F, fabellofibular ligament; popliteofibular ligament; A, arcuate ligament

Another key finding of this study is the impact of the lateral hinge fracture line extension pattern on clinical outcomes. A fracture line extending proximal to the proximal tibiofibular joint is associated with significantly better outcomes compared with other patterns. A possible explanation is that most strong soft-tissue stabilizers of the lateral and posterolateral knee—including the iliotibial (IT) band, lateral collateral ligament, biceps femoris, fabellofibular ligament, popliteofibular ligament, and popliteus muscle—attach proximal to the proximal tibiofibular (PTF) joint, except for the PTF ligaments [19]. In type B and C fractures, these key stabilizing structures remain above the fracture level (Fig. 2). Additionally, since the posterior PTF ligaments are weaker, the PTF joint becomes highly unstable and less capable of handling torsional stress. This instability leads to micromotion, causing delayed fracture union. However, as indicated by Takeuchi et al. [1], type A fractures are proximal to the PTF joint and benefit from the dense and solid connective tissue support, which provide an anatomical advantage for healing (Fig. 2). Type D fractures are unstable because the proximal fragment is supported only by the TomoFix plate. Detecting and visualizing this fracture pattern as early as possible using MRI is crucial, underscoring the importance of MRI in the postoperative classification of lateral hinge fractures. There was no significant difference observed among type A and B in terms of loss of correction and other complications.

This study has several limitations: This retrospective study involved cases where all patients underwent biplanar osteotomy, which may influence fracture incidence and complications compared with uniplanar osteotomy. Only the TomoFix plate and its instruments were used for the surgical procedures, limiting the generalizability of our results to other techniques. Additionally, since all surgeries were performed by a single surgeon, there is a possibility of performance bias.

The authors recommend a postoperative MRI to screen and classify lateral hinge fractures. Type B and C fractures should be managed using a protocol similar to Takeuchi type II. Additionally, they suggest performing a thorough anterolateral osteotomy [20], utilizing the lateral crossing (criss-cross) K-wire method [21, 22] while opening the wedge, and using a 4.5-mm hinge screw in either an anterograde or a retrograde fashion [23–25]. These measures help prevent type B and C fractures and mitigate their impact if they occur.

## Conclusions

According to Takeuchi’s classification, types A and B are both classified as type I. However, type B fractures demonstrated longer union times and a higher risk of delayed union, clinically resembling Takeuchi type II fractures. Type A fractures showed union rates similar to those in nonfracture groups. Of type B fractures detected on immediate postoperative MRI, 90% will become evident only on follow-up radiographs, and their management should be approached with similar concerns to those for Takeuchi type II fractures. This study validates that a variant/subtype of Takeuchi type I fracture has an outcome similar to that of Takeuchi type II fractures and the establishment of distinct rehabilitation protocols for type B fractures.

## Data Availability

The datasets generated and analyzed during the current study are available from the corresponding author on reasonable request.

## Abbreviations

MOWHTO: Medial opening wedge high tibial osteotomy
HTO: High tibial osteotomy
LHF: Lateral hinge fracture
TCP: Tricalcium phosphate
MRI: Magnetic resonance imaging
CT: Computed tomography
